# An Evaluation of the Safety of Intravenous Injections of the Natural Extracellular Hemoglobin M101 in Dogs and Monkeys

**DOI:** 10.3390/ijms26020842

**Published:** 2025-01-20

**Authors:** Elisabeth Leize-Zal, Leïla Demini, Benoît Barrou, Franck Zal

**Affiliations:** HEMARINA S.A., Aéropôle Centre, 29400 Morlaix, France; elisabeth.leize-zal@hemarina.com (E.L.-Z.); leila.demini@hemarina.com (L.D.); benoit.barrou@hemarina.com (B.B.)

**Keywords:** hemoglobin, blood substitute, transfusion, large mammals

## Abstract

Hemoglobin-based oxygen carriers have been developed to compensate the needs of blood for transfusions. Most of them were based on intracellular hemoglobin extracted from bovine or human blood, but unfortunately, this type of hemoglobin did not pass through the last steps of clinical trials. In this context, HEMARINA discovered a natural extracellular hemoglobin, possessing several advantages avoiding intracellular hemoglobin-related side effects. Many preclinical studies assessed the safety of M101 used in intravenous (IV) injection in rodents. To explore the safety of IV injections of M101 in large mammals, six dogs received each a single injection of liquid M101 according to a dose escalation with a 48 h follow-up. Then, two monkeys received multiple IV injections of the same dose of M101 every hour for seven hours. This study showed that single and multiple IV injections in dogs and monkeys did not cause clinical or histological lesions, nor did they induce immunological reactions. This makes M101 the best candidate to date for human use in emergency situations requiring blood and, in several diseases, causing hypoxia problems.

## 1. Introduction

In 2017, a modeling study involving 195 countries and territories estimated that the global need of blood product units (red blood cells or platelets) was around 304,711,244 and the global blood supply was 272,270,243, with a need-to-supply ratio of 1.121. This study demonstrated that the gap between need and supply is large in many low- and middle-income countries and reinforces that the WHO target of 10–20 donations per 1000 inhabitants is an underestimate for many countries [1]. The demand for blood is huge and exceeds the ability of the blood banks to supply it. In addition, blood transfusions meet several limitations: special storage requirements, limited shelf life, infectious risks (HIV, hepatitis), hemolytic and immunogenic complications and poor portable availability in emergency places [2]. The use of hemoglobin solutions for human transfusion met several issues. Patients had anaphylactic symptoms, severe renal toxicity and hypertension [3]. Hemoglobin is essential for O_2_ and CO_2_ transport. In humans, the hemoglobin is intracellular, in the red blood cell (RBC). The RBCs protect hemoglobin from denaturation and oxidation [4]. Oxidation is the change of Fe^2+^ in Fe^3+^ which is unable to fix the oxygen molecules. Outside the RBCs, the hemoglobin becomes unstable and dissociates rapidly into dimeric and monomeric compounds, acting as intravascular and extravascular nitroxide (NO) scavengers [5]. The suppression of NO, which acts as a natural potent vasodilator, results in vasoconstriction and hypertension, stroke and infarction and explains the failure of the trials which aimed to replace human red blood cell transfusions with free human or bovine hemoglobin [6]. The stability and oxygen release of the hemoglobin depend on allosteric effectors such as protons, CO_2_ or 2,3-diphosphoglycerate (2,3-DPG). Indeed, many efforts have been made to replace RBC transfusions by artificial hemoglobin-based oxygen carriers (HBOCs) [7]. HBOCs have several advantages, such as a longer storage duration, decreased or eliminated infectious risk and lack of compatibility issues [8]. Modifications have been made to these HBOCs to reduce the risk of dissociation such as polymerization, cross-linking, conjugation with antioxidant enzymes to reduce the oxidation of the heme iron, and to decrease interactions of HBOCs with the endothelium, thus reducing the NO scavenger effect. Only one bovine hemoglobin, Hemopure^®^, has obtained approval for clinical use in South Africa, in 2001, and is available in the United States only through the Expanded Access Program [7].

In order to circumvent the side effects observed with intracellular hemoglobins, interest in the use of extracellular hemoglobin as a blood substitute appeared a century ago [9]. A giant extracellular hemoglobin has been found in annelids and vestimentiferans [10]. Specifically, the *Arenicola marina* hemoglobin, called M101, is particularly interesting for human use because of its intrinsic properties. The quaternary structure of M101, described by Zal and collaborators, showed that it is made of 156 globin chains with 1 heme molecule and 1 iron molecule in each chain, making M101 able to carry 156 oxygen molecules (40 times more than human hemoglobin (HbA) [11]. Although it is a giant hemoglobin, 60 times bigger than HbA, it is 250 times smaller than RBCs and can, for example, pass into thrombotic vessels and reduce hypoxia in poorly vascularized tissues [11]. M101 has a molecular weight of 3600 kDa with a size of 25 nm and dissociates into dodecamers with a size of around 9 nm and a molecular weight of 205 kDa, large enough to enable it to remain in the vascular compartment and reduce renal clearance, thus preventing the elimination of the molecule [11]. Thanks to its low P50 of ~7.05 mmHg (37 °C), M101 passively releases the oxygen molecules along with an oxygen partial pressure gradient in the absence of allosteric effectors, providing the environment with just the right amount of O_2_, avoiding problems associated with oxidative damage and the NO scavenger effect seen with other HBOCs [12]. In addition, M101 has intrinsic antioxidant Super Oxide Dismutase (SOD)-like activity, due to the presence of copper and zinc molecules in the linker chains, preventing M101 from oxidation and transformation into methemoglobin [11,12]. Intravenous (IV) injection of M101 does not induce any immunogenic reactions, as its chains are not glycosylated, in contrast to those of the earthworm *Lumbricus terrestris* hemoglobin [12].

A top load IV injection of M101 in rats at a concentration of 600 mg/kg did not reveal any effect on heart rate and mean arterial pressure [11,12]. A second experiment with another model, the hamster dorsal skinfold window chamber, showed, after M101 injection (280 mg/kg), the absence of microvascular vasoconstriction and no significant impact on mean arterial blood pressure, in contrast to other HBOCs developed so far [11,12]. This makes M101 a very interesting candidate as an RBC substitute. In a mice model, the IV injection of M101 revealed that M101 displayed a widespread body distribution for at least 5 h and persisted in the body for approximately 96 h with a half-life of 24 h. M101 remained functional, freely circulating for several hours in vivo. In this model again, no obvious clinical toxic or histological side effects were observed at any time after M101 intravenous injection at a dose of 600 mg/kg [11,12].

The aim of this study was to demonstrate the safety of single and multiple intravenous administration of M101 in large mammals with a short (7 h) or long (48 h) follow-up. M101 was injected in beagle dogs (*Canis lupus familiaris*) and in cynomolgus monkeys (*Macaca fascicularis*) and was reported to be an excellent model for developmental toxicity studies [13].

## 2. Results

### 2.1. Clinical and Biological Parameters Assessment After M101 Injection in Dogs and Monkeys

**Figure 1:**
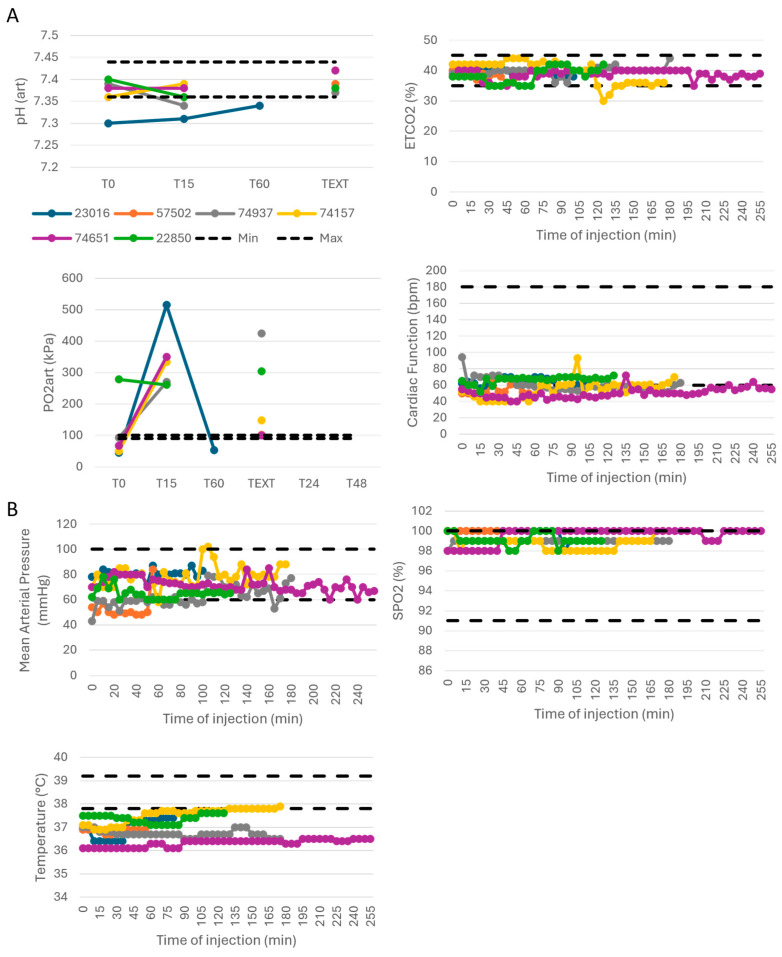
Gasometry and clinical parameters during IV injections of M101 in the six dogs remained in normal range and stable. (**A**) Respiratory parameters. (**B**) Clinical parameters. *n* = 6 for each parameter; each dog is represented individually.

Each of the six dogs received a single injection of M101 according to a dose escalation from 0.5 to 3.032 mg/kg. Clinical parameters were stable during anesthesia and injection of M101 (Figure 1). The subjects presented stable cardiac function, blood pH, pCO_2,_ pO_2_ and stable arterial pressure and SPO_2_ (Figure 1A,B), confirming the absence of any effect on respiratory function and aerobic metabolism throughout the experiments. In all animals, a slight hypothermia was observed due to anesthesia. In two dogs (#22850 and #74157), an increase in temperature of less than one degree was observed during anesthesia, but the temperature remained within the normal range and this slight rise was therefore not considered to be fever (Figure 1B).

**Figure 2:**
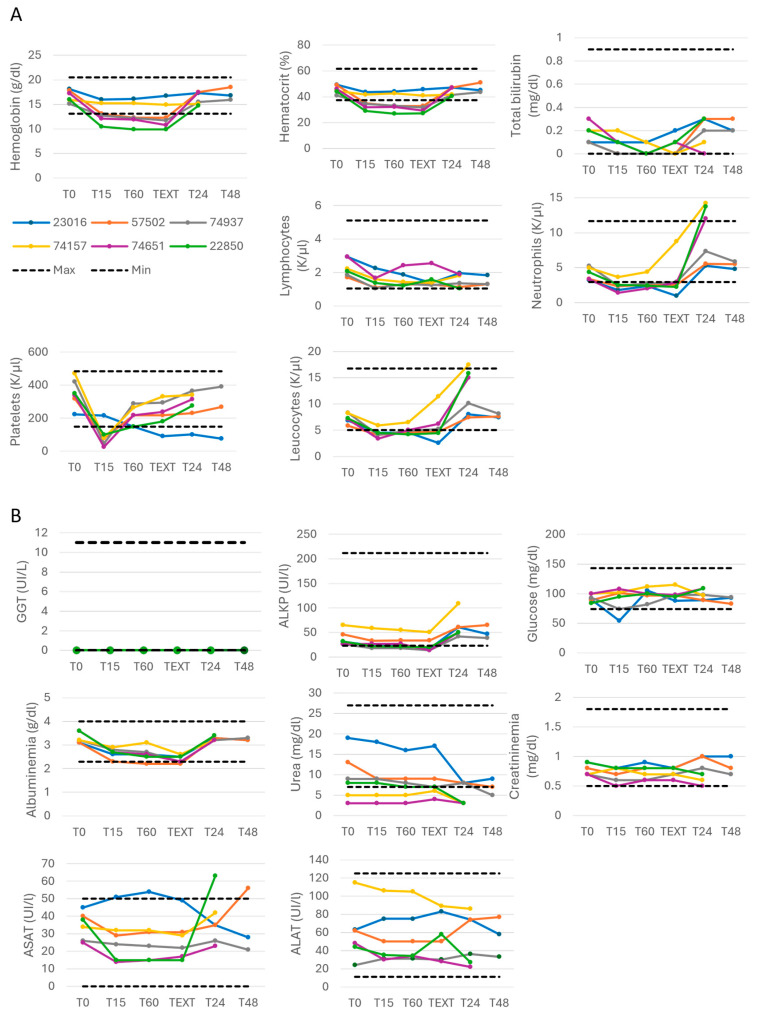
Biological parameters during IV injections of M101 in dogs remained in normal range and stable. (**A**) Blood count. (**B**) Biochemical parameters. *n* = 6 for each parameter; each dog is represented individually.

The blood count evaluating the hemoglobin, hematocrit, bilirubin and immunological cell levels remained stable during the M101 injections in dogs, except for an initial and transitory decrease in platelets count observed (right after the M101 injection) which returned to normal within the first hour (Figure 2A). The ionogram (Supplemental Data S1) and biochemical parameters monitored as γGT, ALKP, glycemia, albuminemia, urea, creatininemia, ASAT and ALAT remained unchanged during the experiment (Figure 2B). The post injection follow-up showed no alteration in the clinical status of the dogs. The different physical exams were normal in terms of mucosa color, capillary refill time, lymphoid nodes, hydration status, cardiac and respiration auscultation, abdominal palpation, pulse, temperature, skin, feces, urine, vomiting and mental behavior. In one dog (#57502, 0.5 mg/kg), the injection had to be stopped after 60 min because of a low and unstable heart rate and diastolic pressure. These two effects were related to intolerance of anesthetics and/or analgesics since they started before M101 administration.

**Figure 3:**
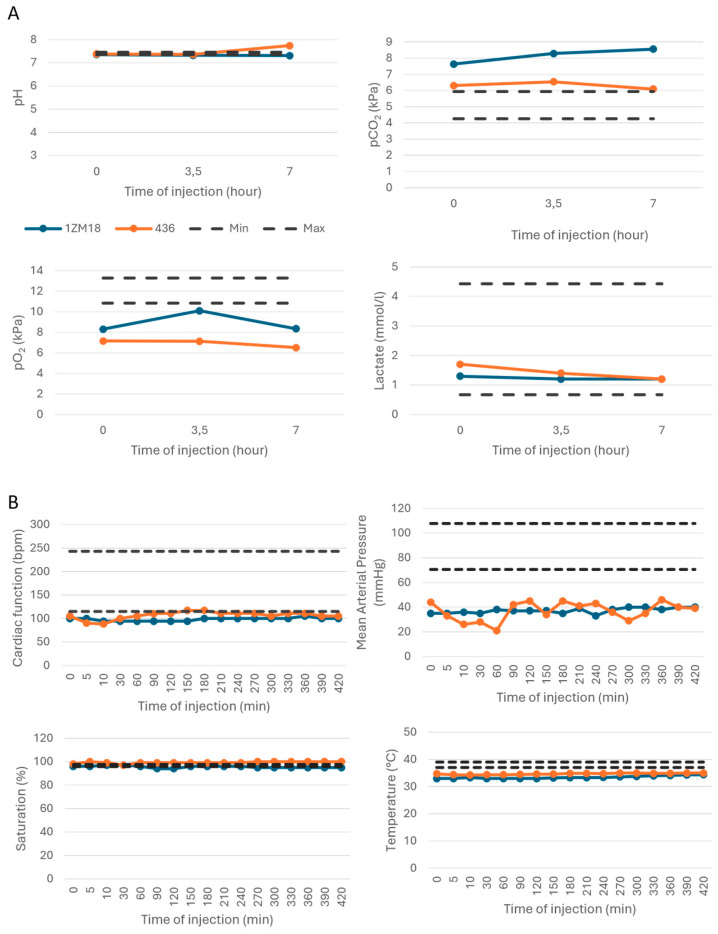
Gasometry and clinical parameters during IV injections of M101 in the two monkeys were normal and stable. (**A**) Respiratory parameters. (**B**) Clinical parameters. *n* = 2 for each parameter; each monkey is represented individually.

The injection of the molecule at a dose of 0.72 mg/kg/h at an hourly bolus (total dose 5.04 mg/kg) was achieved in two primates. As in dogs, the subjects presented stable blood pH, pCO_2_, pO_2_ and lactate levels throughout the experiment, confirming the lack of effect on respiratory functions and aerobic metabolism. Respiratory alkalosis related to anesthesia and intubation may be noted with a slight increase in the pH for the monkey #436 (Figure 3A). Clinically, both subjects presented stable clinical parameters during the experiment (heart rate, blood pressure, saturation) (Figure 3B).

**Figure 4:**
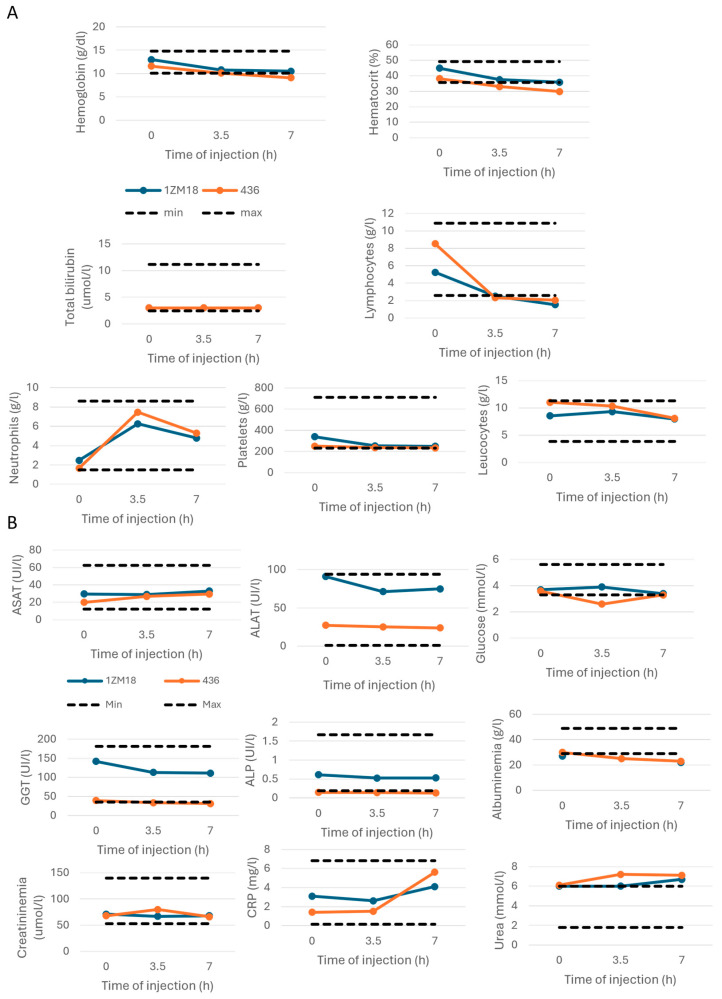
Biological parameters during IV injections of M101 in monkeys were normal and stable except for the transitory increase in neutrophils and decrease in lymphocytes. (**A**) Respiratory parameters. (**B**) Clinical parameters. *n* = 2 for each parameter; each monkey is represented individually.

As in dogs, the temperature was lowered due to anesthesia, despite the placement of a heating blanket on the animal (Figure 3B). The blood count evaluating the hemoglobin, hematocrit, bilirubin and immunological cell levels remained stable during the M101 injections in monkeys (Figure 4A). We observed a decrease in native hemoglobin levels during the experiment (Figure 4A). Stability of the leukocyte count was observed during the experiment. However, a slight decrease in the lymphocyte count was associated with a transitory increase in the neutrophil count. Both values remained however in the normal ranges (Figure 4A). Within the lymphocyte population, the respective proportions of CD4+ T cells, CD8+ T cells, B cells and NK lymphocytes remained unchanged (Supplemental Data S2A). The ionogram (Supplemental Data S2B) and biochemical parameters monitored as ASAT, ALAT, glycemia, γGT, ALKP, albuminemia, creatininemia, CRP and urea remained unchanged during the experiment (Figure 4B). The clinical and biochemical analyses during the injections of M101 in dogs and monkeys were in favor of an absence of toxicity related to intravenous M101 injection.

### 2.2. Histological Analysis of Selected Organs After M101 Injection in Dogs and Monkeys

**Figure 5:**
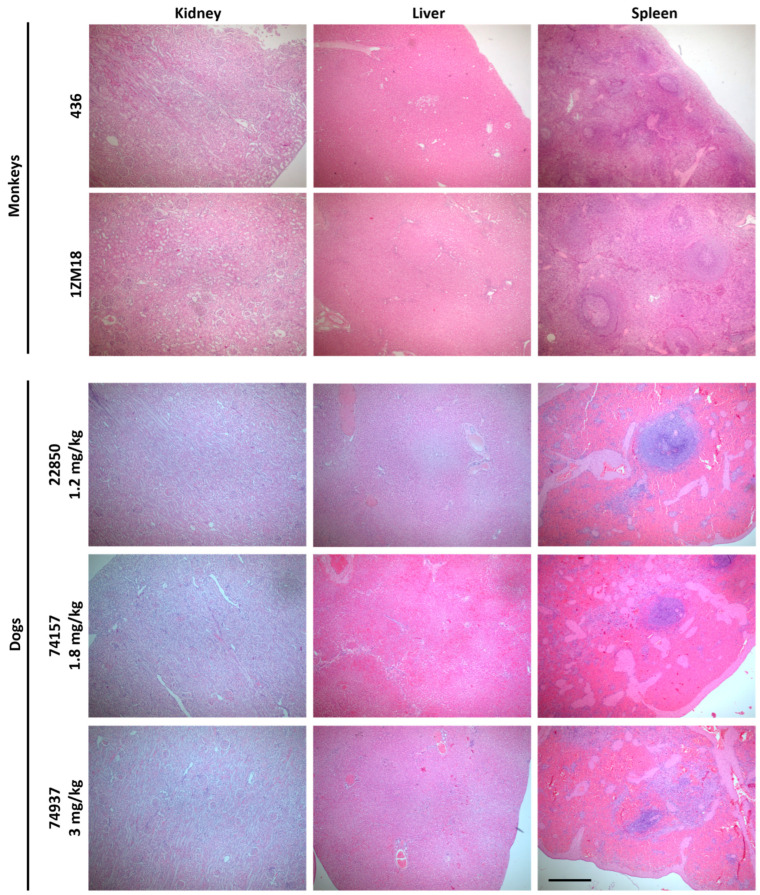
Histological analysis of kidney, liver and spleen after IV injections in both monkeys (**up**) and dogs (**down**) did not show any toxic effect after IV M101 injection. Histological slides were colored by hematoxylin and eosin and observed using a light microscope (scale bar = 0.25 cm).

In dogs and monkeys, necropsies did not reveal macroscopic pathologic lesions in the organs after the injection of M101 (Figure 5). For both species, the macroscopic lesions reported, like congestive kidneys, sparkling content in ileum, splenomegaly, pulmonary congestion and edema with foamy fluid in the trachea and bronchi, are compatible with the method of euthanasia. Histological analyses were performed by two independent histopathologists, one being blinded. They revealed no microscopic signs of toxicity related to the M101 injection in any of the organs and confirmed that the macroscopic lesions observed were compatible with the use of barbiturates during the euthanasia procedure [14].

### 2.3. Complement, Allergy, Inflammatory and Humoral Pathways Reaction to M101 Intravenous Injection

**Figure 6:**
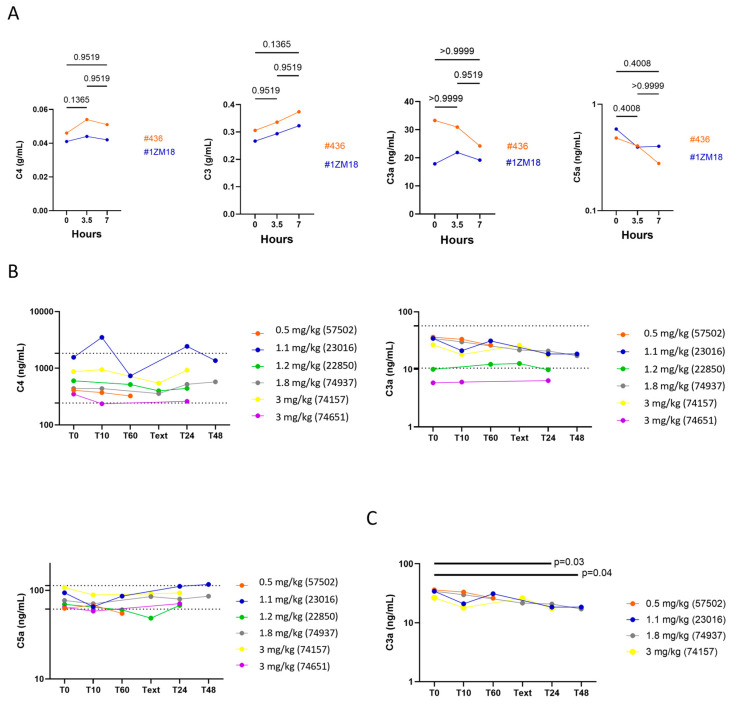
M101 injections did not activate the complement pathway in both species and may inhibit the C3a release. The complement pathway was analyzed by measuring the C3, C3a, C4 and C5 molecules levels by ELISA assay in monkeys (*n* = 2) (**A**) and dogs (*n* = 6) (**B**). In a sub-analysis, a significant difference (ANOVA *p* = 0.04) was observed at T24 and T48 when dogs with C3a levels equal or below 5 ng/mL at basal level (T0) were removed from the analysis (*n* = 4) (**C**). *p* values under 0.05 were considered significant.

To evaluate if M101 induces an immunological reaction during the injections, we measured the activation of the complement pathway, the allergy pathway, the humoral pathway and pro-inflammatory cytokines. The assessment of the complement activation showed that the injections of M101 did not induce the complement activation pathway in dogs nor in monkeys (Figure 6A,B). In dogs, an inhibition of the basal level of the C3 can be suspected (Figure 6C).

**Figure 7:**
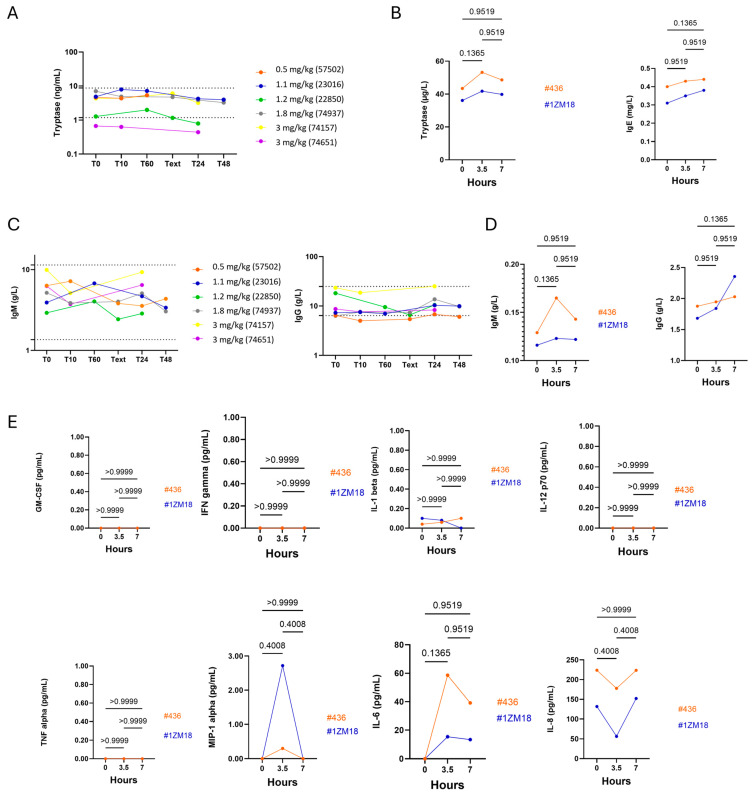
Allergic, humoral and inflammation pathway and cytokines release analysis in dogs and monkeys showed that IV injections of M101 did not induce allergic and inflammatory reactions. The allergy pathway was analyzed by measuring the tryptase ((**A**) in dogs) and the tryptase and the IgE ((**B**) in monkeys) levels by ELISA assays. The IgM and IgG were measured by ELISA assays to analyze the humoral response in dogs (**C**) and monkeys (**D**). Inflammatory cytokines’ release was measured by multiplex luminex assay in monkeys (**E**). *p* values under 0.05 were considered significant. *n* = 6 for dog experiment, *n* = 2 for monkey experiment.

To assess the activation of the allergy pathway, we measured the tryptase levels, which are released by mast cells during an acute allergic reaction. The allergy pathway can be induced by lgE-dependent or -independent mechanisms, and both mechanisms are clinically indistinguishable. Measurement of tryptase in both dogs and monkeys showed that circulating tryptase levels were stable over time and IgE levels were not affected in monkeys after intravenous injection of M101 at 3.5 and 7 h compared to baseline. Therefore, M101 did not activate either tryptase or IgE allergic pathways in the monkeys (Figure 7A,B). Measurements of the quantitative antibody response (lgM and lgG) have been used in evaluating the humoral immune response (Figure 7C,D). No changes were observed in dogs in IgM and IgG levels. However, an unexplained increase in lgG level from baseline at 7 h in monkeys has been observed (Figure 7D). This effect was not reported for lgM and lgE and may result from intra-assay variations. Analysis of inflammatory cytokines by ELISA assay showed that circulating levels of TNF-alpha and IFN-gamma in dogs were below the detection limit from T0 to 48 h after intravenous injection of M101 and did not rise above the detection limit after injection of M101. In monkeys, the GM-CSF, IFN gamma, IL-1 beta, IL-12p70 and TNF alpha cytokines were below the limit of detection at the three time points (Figure 7E). We observed a transitional and non-significative cytokine release for MIP-1 alpha and IL-6, which was not surprising as MIP-1 alpha acts upstream IL-6 in the inflammatory cascade. Return to the baseline was observed at 7 h for MIP-1 alpha and almost achieved for IL-6. We cannot exclude that this transient increase in IL-6 may result from the local inflammatory response due to the insertion of the intravenous catheter and/or the presence of LPS traces on it.

## 3. Discussion

The use of intracellular hemoglobin as a blood substitute was associated with several side effects such as hypertension, vasodilatation, oxidation, etc. In this study, we show that animals treated with intravenous infusion of M101, an extracellular natural hemoglobin, showed no side effects either on blood hemodynamic or blood analyses. The macroscopic aspect of the organs revealed no effect related to M101 injection. At the clinical level, both species presented stable clinical constants during the experiment (heart rate, blood pressure, saturation). Although lesions have been observed during microscopic analysis mainly in kidneys, a double analysis by histopathologists, including one blinded, concluded that these lesions were due to the method of sacrifice. Indeed, the use of barbiturates overdose is known to cause liver and kidney damages associated with increased levels of HIF1-α and TNF- α and that they may interfere with the biochemical, molecular and histological measurements [14]. It was observed that M101 did not induce an immunological reaction. However, a slight decrease in the C3 level was observed, consistent with in vitro investigations on human blood samples showing that M101 can inhibit the C3 convertase [15]. A decrease in the hemoglobin levels is observed after IV administration. One hypothesis is that it may inhibit the production of endogen hemoglobin to maintain a homeostatic hemoglobin level. Of note, the method for Hb dosage in dogs and monkeys uses species-specific antibodies that are therefore unable to detect M101 which is a natural extracellular and not glycosylated hemoglobin. This inability to detect M101 by usual antibodies can be of concern, as M101 is a promising substitute for RBC transfusion but could also be misused by cheating athletes. However, a recent modified approach of an existing doping control detection method for bovine HBOCs was able to specifically detect M101 at a sensitive level (down to concentrations of 10 μg/mL from 50 μL of serum/plasma) [12]. M101 could be detected 8 h after injections in a rat model, which is reassuring in case of misuse of the molecule. Many HBOCs have been developed from intracellular hemoglobin but with different modifications to prevent adverse effects due to the natural intracellular, and thus protected, location. Indeed, acellular hemoglobin extracted from RBCs, mostly from human or bovine blood, is unstable and rapidly cleared by kidneys. Its affinity for oxygen is no longer regulated by allosteric cofactors such as 2,3-DPG, resulting in a dysregulated tissue oxygenation capacity. In addition, Hb molecules dissociate rapidly once extracted from the RBC and act as a potent scavenger of nitric oxide (NO), resulting in vasoconstriction effects, which probably explain the side effects observed in the clinical setting, such as hypertension, stroke and infarction. Acellular hemoglobin solutions are also very sensitive to irreversible oxidation into methemoglobin, losing its ability to transport oxygen [6]. Different types of modifications of intracellular hemoglobins have been proposed: polymerization increasing the hemoglobin size and half-life, cross-linking the two alpha and beta chains either with raffinose or diaspirin, to stabilize the molecule and prevent its excretion by kidneys; conjugation with antioxidant molecules to prevent the free radical negative effects (increasing, by the way, the size of the molecule, thus reducing its glomerular filtration and the NO scavenger effect; and encapsulation into phospholipid bilayer capsules, with the idea to reproduce the protection of the RBC membrane. Unfortunately, even if most of the modified acellular hemoglobin solutions have reached clinical phases, none were able to be commercialized and to pass through the last steps of clinical studies. Furthermore, pegylated protein, such as some manufactured HBOCs, after IV injection could provoke immune reactions [16].

In this context, HEMARINA has developed a new approach to overcome the obstacles inherent in the intracellular nature of the hemoglobin used to date. M101, the bulk product of HEMARINA, a breakthrough innovation, is the purified natural extracellular hemoglobin extracted from the polychaete annelid *Arenicola marina* (M101) in GMP conditions. As M101 is naturally extracellular, none of the side effects observed after intracellular Hb administration are seen after M101 administration. Many applications have been developed using M101. HEMO2life^®^, a class III medical device containing 1 g/L of M101, is used in renal transplantation (HEMARINA, Morlaix, France). A national multicenter open-label study (OXYOP study; Clinical Trial Registry No. NCT02652520) was designed to evaluate the safety and performance of HEMO2life^®^ used ex vivo as an additive to the preservation solution in kidney transplantation [17]. This study demonstrated the safety for both graft and kidney recipients and the performance measured by the Delayed Graft Function rate, which was significantly decreased in the HEMO2life^®^ group 1-year post-transplant. The patient follow-up (Clinical Trial Registry No. NCT05050513) at 4 years post-transplant demonstrated a significantly better patient survival rate using HEMO2life^®^ (98.3% HEMO2life^®^ arm vs 86% Control arm) and the absence of immunological reactions, demonstrating for the first time the benefit of using an oxygen carrier for organ preservation [12]. M101 has been used for graft preservation in a human kidney transplant clinical trial. Grafts were placed in standard preservation solutions containing 1 g/L of M101, rinsed at the end of the preservation period and transplanted. There were no reported side effects associated with the use of M101 [17]. However, a lower DGF rate and a better patient survival at 4 years were observed using M101 during the preservation [12]. M101 has also been used in a human face retransplant case. The face—a composite tissue graft—was perfused and then immersed in IGL-1 preservation solution (IGL, Lissieu France) containing 1 g/L of M101. In this case, the graft was not rinsed prior to transplantation. Plasma samples were taken 15 and 30 min after transplantation. FPLC analysis detected M101 at a concentration of 0.044 g/L in the patient’s blood (unpublished data). No toxicity related to the M101 molecule was reported. M101 has also been used in three additional cases of composite tissue transplantation in humans: one case of face transplantation [18] and two cases of arm transplantation without side effects linked to M101 use. Unpublished internal data (registered as internal and official documents under our Quality Management System) demonstrated that M101 did not induce platelet aggregation nor cytokine reactions in vitro on human blood samples. The present study as well as previous preclinical and clinical evidence (mostly summarized in [11]) demonstrate that M101 can be safely administrated intravenously in mammals. M101, in its IV form, called HEMOXYCarrier^®^, could be used in different hypoxic situations in the clinical setting, such as vaso-occlusive crisis in patients with sickle cell disease, strokes, hemorrhagic shock, etc. M101 IV injection could also be a crucial way to improve cancer treatments. Indeed, a hypoxic environment is created in the core of tumors, decreasing the efficacy of radiotherapy, which uses the oxygen to generate ROS to kill cancer cells. A study showed that in a mouse model injected with different types of subcutaneous tumors, a single IV injection of M101 increased the oxygenation of the tumor [11,12]. This was visualized by a decrease in Glut-1 staining, a marker of hypoxia, and this effect was very fast, as it occurred only 15 min after injection. This demonstrates the ability of M101 to diffuse within poorly vascularized tissues. The ability of M101 to carry oxygen in hypoxic tissues was also investigated by injecting M101 in rats after cardiac arrest and during cardiopulmonary resuscitation. This study showed that M101 had a protective effect and decreased hypoxic brain injuries [19].

Recently, HEMARINA developed a lyophilized form of the M101 molecule containing all ion elements. After resuspension with ppi water, this form was injected in rats. After 72 h of monitoring, the rats did not show any differences in terms of behavior or pain. The macroscopic and histological analysis after the sacrifice of the rats did not show any disturbance in the hepatic, cardiac and hemostatic functions, indicating no impairment of hepatic and cardiac function (manuscript in preparation). This lyophilized form of M101 will make the molecule more readily available in emergencies at accident sites and will considerably extend the product’s shelf life, providing a solution to the many logistical constraints of emergency medicine in terms of urgent RBC transfusions. In addition, as M101 does not display the surface Ag of RBC, it can be regarded as the first universal hemoglobin.

The present study shows the safety of IV injections of M101 in monkeys and dogs for 7 h and 48 h, respectively. To confirm the full safety of the molecule, we agreed that a longer time period should be considered in vivo. This was achieved with injections in dogs, which were monitored for 48 h after IV injection. The number of monkeys and dogs used in this study was determined in agreement with ethics committees since the data already obtained during human transplantation of organs and vascular tissue composites had demonstrated no side effects. Altogether, the results of this study, with the previous preclinical and clinical studies, show that M101 is safe for IV use in large mammals and can now be tested intravenously by clinical trials in humans.

## 4. Materials and Methods

### 4.1. Intravenous Injections of M101

For both species, Ringer solution was supplemented with a liquid form of M101 at a final concentration of 1 g/L and both species received M101 solution by intravenous top load injections.

#### 4.1.1. In Dogs

Six female beagle dogs were supplied by Isoquimen SL (Barcelona, Spain). The protocol was approved by the Institutional Animal Ethics Committee of the Rof Codina Foundation (Approval number: 01/21/LU-001). The study was performed by iBoneLab SL (Lugo, Spain) in a dedicated facility (CEBIOVET, Lugo, Spain). Dogs were sedated with a combination of intramuscular (IM) medetomidine 10 µg/kg (Sededorm^®^ 1 mg/mL, Vetpharma Animal Pharmaceutical Company, Barcelona, Spain) and butorphanol 0.1 mg/kg (Butomidor^®^ 10 mg/mL, Richter Pharma, Wels, Austria). General anesthesia was induced by intravenous injection of propofol 2 mg/Kg (Propofol Lipuro^®^ 10 mg/mL, B Braun, Melsungen, Germany) for orotracheal intubation and maintained with Isoflurane 1–1.5% in 100% O_2_ (Vetflurane^®^ 1000 mg/g, Virbac SA, Carros, France) with mechanical ventilation. The following clinical parameters were monitored during the experiment: heart rate; respiratory frequency (mechanical ventilation); expiratory rate of CO_2_ (ETCO_2_); systolic, diastolic and mean blood pressure; O_2_ saturation (SPO_2_); and temperature. For the entire procedure, animals were covered by an emergency blanket to prevent hypothermia. Also, a bag of warm water was placed in contact with the animal and changed when necessary. The values were recorded initially every 5 min during the first 30 min of infusion and then every 15 min if parameters were normal. If not, values were recorded every 5 min again. Each of the six dogs received a single injection of liquid M101 (batch number S208/ORGAN/FC002) according to a dose escalation, from 0.5 to 3.032 mg/kg, with a maximum flow of 10 mL/kg/h. If, during the infusion of one animal, no side effects were observed, then the increased dose was administered to the next animal. This approach was designed to find the highest dose of M101 without side effects. After the infusion, animals were housed in controlled conditions of temperature, humidity and ventilation. The animals were fed with commercial granulated dog food and had free access to water. The clinical status of dogs was monitored twice per day during the entire experiment by the veterinarian and facility staff. Blood samples were collected just before M101 infusion (T0), 10 or 15 min after the beginning of M101 infusion (T10 or T15), 60 min after infusion initiation (T60), at the end of infusion, after extubation (T-ext), 24 h post infusion (T24) and 48 h post infusion (T48). Animals were sacrificed with an intravenous overdose of Sodium Pentobarbital (Dolethal^®^ 200 mg/mL, Vetoquinol Especialidades Veterinarias SA, Madrid, Spain). Animals were then taken to the Pathology unit for a complete and systematic necropsy. Organs were then collected in Formalin 10% for further histological analysis.

#### 4.1.2. In Macaques

Two cynomolgus monkeys were used in the study after the approval of the protocol by the ethical committee (APAFiS# 35-973). For ethics reasons, it was decided to use a small number of macaques for this experiment, but of both sexes, one male and one female, because hematological and biochemical parameters depend on sex [20]. The procedures and animal housing were performed by Le Laboratoire des Grands Animaux, University Hospital Nantes, INSERM UMR1064, Nantes, France. One hour before experiments, macaques were placed under general anesthesia. After sedation with Zolétil (Zolazepam/Tiletamine) at 15 mg/kg IM (0.2 to 0.5 mL), anesthesia was induced by a mask-mixture of 2% isoflurane, 49% nitrous oxide and 49% oxygen before the placement of an intubation cannula. Anesthesia was maintained by the administration of the same gas mixture at an insufflation volume of 10 mL/kg. Several parameters were monitored during the experiment: systolic, diastolic and mean arterial blood pressure; saturation in O_2_ (SPO_2_) and heart rate (monitored by a pulse oximeter); respiratory function (respiratory frequency, expiratory and inspiratory rate in CO_2_); and the temperature. Injections of M101 were performed through the left femoral central venous line set up surgically. M101 in its liquid form (batch number E0217MOL0119) was injected at a dose of 0.72 mg/kg every hour for seven hours (7 injections, total dose 5.04 mg/kg). Blood samples were collected at T = 0, T = 3.5 h and T = 7 h. At the end of the procedure, the animals, while still under general anesthesia, were euthanized by an overdose of pentobarbital (140 mg/kg) (Dolethal^®^, Centravet, Paris, France). Organs were then collected in Formalin 10% for further histological analysis.

### 4.2. Biological Analysis

#### 4.2.1. In Dogs

The following blood parameters were analyzed using Procyte One (Catalyst one, VetStat, IDEXX, Westbrook, ME, USA): complete blood count, albumin, alanine aminotransferase (ALAT), Aspartate aminotransferase (ASAT), Phosphatase alcaline (ALKP), total bilirubin, cholesterol, serum creatinine level, gamma glutamyl transferase (γGT), glucose, total protein, triglycerides, immunoglobulins, ionogram (Ca, Cl, P, K, Na) and blood gases.

#### 4.2.2. In Macaques

The blood count, biochemistry, hemostasis and blood gases were analyzed by the University Hospital of Nantes analysis platform (Nantes, France). The immunomonitoring was performed by Fluorescence-activated Cell Sorting (FACS CantoII, BD Bioscience, Grenoble, France) and the results were analyzed with FlowJo software. The antibodies used for the FACS analysis are listed in Table 1.

### 4.3. Immunological Parameters Analysis

The plasma collected from the six Beagle dogs were stored at −20 °C in heparin tubes until analysis. The peripheral blood sera collected from the three macaques were stored at −80 °C in dry tubes until analysis. The complement pathway (C3 total, C4 total, C3a, C5a), the humoral pathway (IgG, IgM, IgE) the allergy pathway (tryptase) and inflammation (tumor necrosis factor TNF alpha, dogs only), the memory T and the NK cells’ activation (interferon IFN gamma, dogs only) were analyzed using ELISA kits, listed in Table 2.

In monkey experiments, the pro-inflammatory cytokines GM-CSF, IFN-gamma, IL-1 beta, IL-6, IL-8, IL-12p70, MIP-1 alpha and TNF-alpha were analyzed using the multiplex luminex assay (Procarta 8-Plex, PPX-08-MX47XU, Thermofisher, Illkirch, France) (Table 3).

For statistical analysis, data were paired, a non-parametric test was used (Kruskal–Wallis), multiple comparisons were performed, and a post hoc Dunn correction was applied. *p* values under 0.05 were considered significant. All tests and figures were built with Prism 9.5 (GraphPad Software, La Jolla, CA, USA).

### 4.4. Histological Analysis

Sample preparation and histological analyses were performed by histopathologists at iBone Lab SL (Lugo, Spain) in accordance with International Organization for Standardization (ISO) 10993: Biological evaluation of medical devices—Part 11: Tests for systemic toxicity (ISO 10993-11:2017) [21]. At sacrifice, the following organs were collected and weighed: heart, liver, adrenal glands, kidneys, rate, brain, ovaries or testes, epididymis, uterus and thymus. Histological analysis was performed on the following organs: heart, liver, adrenal glands, kidneys, skin, spleen, muscle, brain, ovaries or testis, lungs, bronchi, femur and macroscopic lesions if applicable (including injection site). The blocks containing the samples were prepared following the routine methods for inclusion in paraffin, except for the femur, which was previously decalcified in a solution of hydrochloric acid. Briefly, the blocks were dehydrated in different graded ethanol series (70–100%), defatted in xylol and infiltrated with paraffin. Once polymerized, blocks were sectioned using a microtome and slides were dried and stained with H&E for analysis. Images were obtained using a light microscope and a digital camera connected to a PC-based image capture system (BX51, DP71, Olympus Corporation, Hachioji, Tokyo, Japan).

## 5. Conclusions

This study showed that hemoglobin M101 is the first hemoglobin-based oxygen carrier to induce no toxicity in the various organs analyzed histologically, and no side effects were observed in terms of the clinical, biochemical and immunological parameters monitored during and after intravenous injections in mammals. On the basis of preclinical and clinical studies, hemoglobin M101 would be the best candidate to date for human use in emergency situations requiring blood and in several diseases causing anemia and hypoxia problems.

## Figures and Tables

**Table 1:** Antibodies used to analyze the blood cell count from macaque blood samples by FACS. BD Bioscience, Grenoble, France; BC Beckman Coulter, Indianapolis, IN, USA.

Ab FACS	Clone	Fluorochrome	Supplier	Batch	Dilution
CD3	SP34.2	PECy7	BD: 557749	2059619	1:10
CD4	L200	PerCpCy5,5	BD: 552838	2122303	1:100
CD8	SK1	VB421	BD: 740093	2034316	1:20
CD16	3G8	FITC	BD: 555406	9036589	1:10
CD20	2H7 = L27	APC	BD: 559776	1265292	1:10
NKp46 (CD338)	BAB281	PE	BC: IM3711	200051	1:10

**Table 2:** Antibodies used for immunological analysis in dogs. MBS, İzmir, Türkiye; Abbexa, Leiden, The Netherlands.

	Supplier	Reference	Batch
C4 (total)	MBS	MBS1609519	202209002
C3 (total)	MBS	MBS2501220	AK07X2TZ7498
C3a	Abbexa	abx150230	E2209274W
C5a	Abbexa	abx150231	E2209192U
Tryptase	Abbexa	abx150301	E2209606Y
lgE	MBS	MBS2501977	DP05H8LF7468
lgM	MBS	MBS7607940	CA0080H111
lgG	MBS	MBS8800304	20362324406
TNF-α	MBS	MBS761131	CA0020H116
IFN-γ	MBS	MBS453504	EDL2022112187

**Table 3:** Antibodies used for immunological analysis in monkeys. MBS, İzmir, Türkiye; Thermofisher, Illkirch, France.

	Supplier	Reference	Batch
**ELISA assay**			
**lgG**	MBS	MBS705308	F13142173
**lgM**	MBS	MBS037089	june-23
**lgE**	MBS	MBS033782	june-23
**Tryptase**	MBS	MBS005180	june-23
**C3 (total)**	MBS	MBS9344230	june-23
**C4 (total)**	MBS	MBS020302	june-23
**C3a**	MBS	MBS744614	20230626C
**C5a**	MBS	MBS764389	MK00121066
**Multiplex luminex assay (Procarta 8-Plex)**			
**GM-CSF**	Thermofisher	PPX-08-MX47XU	372421
**IFN-γ**	Thermofisher	PPX-08-MX47XU	372421
**IL-1β**	Thermofisher	PPX-08-MX47XU	372421
**IL-6**	Thermofisher	PPX-08-MX47XU	372421
**IL-8**	Thermofisher	PPX-08-MX47XU	372421
**IL-12p70**	Thermofisher	PPX-08-MX47XU	372421
**MIP-1α**	Thermofisher	PPX-08-MX47XU	372421
**TNF-α**	Thermofisher	PPX-08-MX47XU	372421

## Data Availability

The original contributions presented in this study are included in the article/Supplementary Materials. Further inquiries can be directed to the corresponding author.

## Supplementary Materials

The following supporting information can be downloaded at: https://www.mdpi.com/article/10.3390/ijms26020842/s1.
