# Supplementary material for: An Evaluation of the Safety of Intravenous Injections of the Natural Extracellular Hemoglobin M101 in Dogs and Monkeys
Source: Int J Mol Sci. 2025 Jan 20;26(2):842. doi: 10.3390/ijms26020842 (PMC11765992; doi:10.3390/ijms26020842)
Supplement: Supplementary file 1 [file ijms-26-00842-s001.zip › ijms-3379257-supplementary.pdf]

International Journal of Molecular Sciences

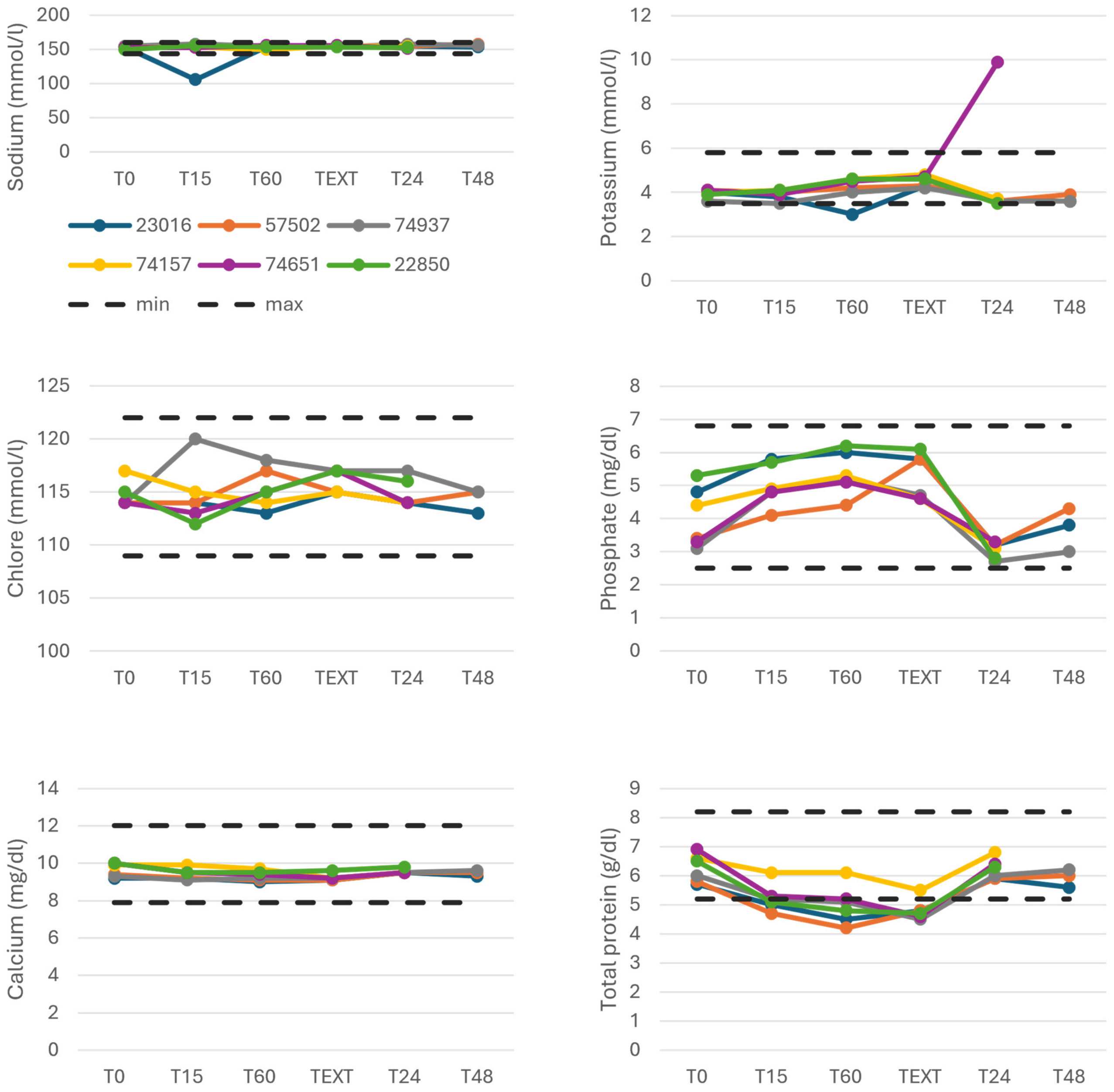

Supplemental Data S1. Ionogram analysis in dogs. $n = 6$.

A

Monkey 1ZM18

Time (hour)	Absolute value en lymphocytes (Giga/L)	Lymphocytes B (%)	Lymphocytes T CD4+	Lymphocytes T CD8+	NK cells (%)
T = 0	5,25	20,40%	20,99%	41,91%	4,34%
T = 3.5h	2,52	15,50%	24,82%	37,37%	5,65%
T = 7.0h	1,54	20,70%	23,56%	30,92%	3,96%

Monkey 436

Time (hour)	Absolute value en lymphocytes (Giga/L)	Lymphocytes B (%)	Lymphocytes T CD4+	Lymphocytes T CD8+	NK cells (%)
T = 0	8,54	17,90%	26,51%	44,54%	1,21%
T = 3.5h	2,34	18,00%	34,23%	40,04%	0,40%
T = 7.0h	2,05	22,80%	29,38%	38,05%	0,25%

B

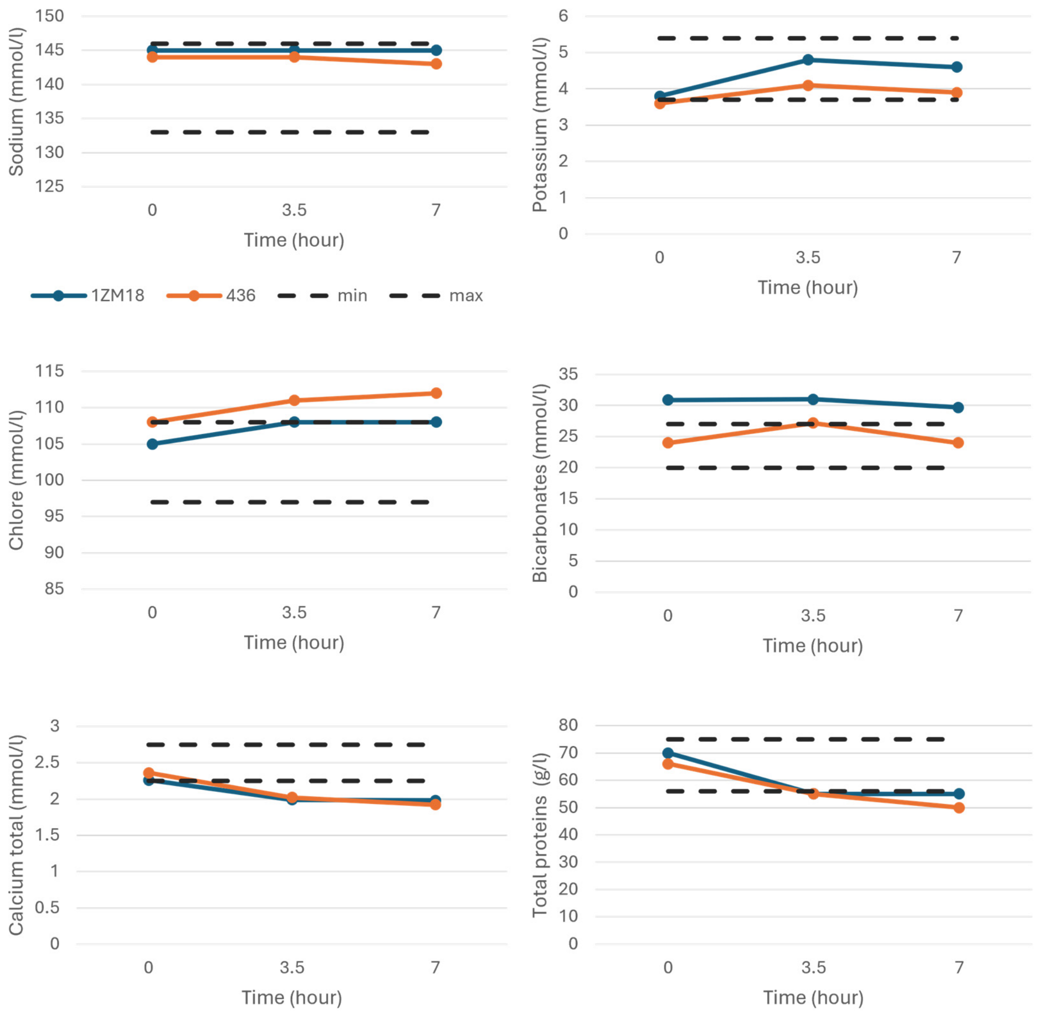

Supplemental Data S2. Immunological and biochemical analysis in monkeys. Percentage of subpopulations relative to the total lymphocyte population analysed by FACS (**A**). Ionogram analysis in monkeys (**B**). $n = 2$.

Disclaimer/Publisher's Note: The statements, opinions and data contained in all publications are solely those of the individual author(s) and contributor(s) and not of MDPI and/or the editor(s). MDPI and/or the editor(s) disclaim responsibility for any injury to people or property resulting from any ideas, methods, instructions or products referred to in the content.
